# Improving protein coreference resolution by simple semantic classification

**DOI:** 10.1186/1471-2105-13-304

**Published:** 2012-11-17

**Authors:** Ngan Nguyen, Jin-Dong Kim, Makoto Miwa, Takuya Matsuzaki, Junichi Tsujii

**Affiliations:** 1 National Institute of Informatics, Hitotsubashi 2-1-2, Chiyoda-ku, Tokyo, Japan; 2 Database Center for Life Science, Yayoi 2-11-16, Bunkyo-ku, Tokyo, Japan; 3 The National Centre for Text Mining, Manchester Interdisciplinary Biocentre, University of Manchester, 131 Princess Street, Manchester, M1 7DN, UK; 4 Microsoft Research Asia, 5 Dan Ling Street, Beijing, Haiian District, China

## Abstract

**Background:**

Current research has shown that major difficulties in event extraction for the biomedical domain are traceable to coreference. Therefore, coreference resolution is believed to be useful for improving event extraction. To address coreference resolution in molecular biology literature, the Protein Coreference (COREF) task was arranged in the BioNLP Shared Task (BioNLP-ST, hereafter) 2011, as a supporting task. However, the shared task results indicated that transferring coreference resolution methods developed for other domains to the biological domain was not a straight-forward task, due to the domain differences in the coreference phenomena.

**Results:**

We analyzed the contribution of domain-specific information, including the information that indicates the protein type, in a rule-based protein coreference resolution system. In particular, the domain-specific information is encoded into semantic classification modules for which the output is used in different components of the coreference resolution. We compared our system with the top four systems in the BioNLP-ST 2011; surprisingly, we found that the minimal configuration had outperformed the best system in the BioNLP-ST 2011. Analysis of the experimental results revealed that semantic classification, using protein information, has contributed to an increase in performance by 2.3% on the test data, and 4.0% on the development data, in F-score.

**Conclusions:**

The use of domain-specific information in semantic classification is important for effective coreference resolution. Since it is difficult to transfer domain-specific information across different domains, we need to continue seek for methods to utilize such information in coreference resolution.

## Background

While named entity recognition (NER) and relation/event extraction are regarded as standard tasks for biomedical information extraction (IE), coreference resolution [1-3] is being recognized more and more as an important component of IE to achieve a higher performance. Coreference structure is so abundant in natural language text, that without properly dealing with it, it is often difficult to capture important information pieces in text. It was also a lesson from the BioNLP Shared Task (BioNLP-ST) 2009, which was a community-wide campaign of bioIE system development and evaluation [4-6], that coreference structures in biomedical text substantially hinder the progress of fine-grained IE. Readers are referred to [4,7] for details about BioNLP-ST. There have been also several attempts for coreference resolution for IE, most of which are focused on newswire texts [3,8-12].

**Figure 1:**
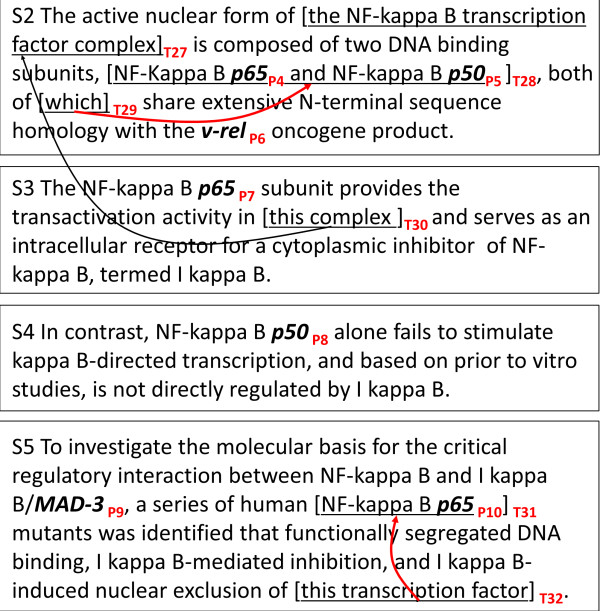
**An excerpt of protein coreference annotated data.** Given protein names are highlighted in purple. Pronouns and definite noun phrases, are highlighted in red, T27, T29, T30, T32, of which the antecedents are indicated by arrows.

To address the problem of coreference resolution in molecular biology literature, the Protein Coreference (COREF) task was arranged in BioNLP-ST 2011. Figure 1 shows an example text segmented into four sentences, S2 - S5, where **coreferential expression**s are shown in brackets. In the task, particularly references to proteins were the primary target of coreference resolution. In the figure, protein names P4 - P10 are highlighted in bold-face. In the example, the definite-noun-phrase expression, *this transcription factor* (T32), is considered coreferential with the protein mention *p65* (P10). Without knowing this **coreference relation**, it becomes difficult to capture the information written in the phrase, *nuclear exclusion of this transcription factor*, which is a *localization of p65 (out of nucleus)*, according to the framework of BioNLP-ST. We call the specific protein reference contained in the antecedent expression, e.g., *p65* in *NF-kappa B p65*, the antecedent protein. Coreferential expressions which do not involve antecedent proteins are out of the focus of the COREF task, e.g., T30→T27. In figure, only the coreferences, T29→T28 and T32→T31 are the target of extraction, as they involve antecedent proteins.

The best-performing system in the COREF shared task found 22.2% of the anaphoric protein references at the precision of 73.3% (34.1% F-score). The system is basically a re-engineered version of existing coreference resolution system that was originally developed for the newswire domain. While the core resolution engine was remained as the same, modifications were mostly made to the processing for markable detection and the post-processing for outputting coreference links according to the task definition [13]. When it was evaluated using the MUC score [14], the system’s performance dropped from 66.38% for newspaper texts to 49.65% for biology texts [13,15], which was perhaps caused by domain differences.

A detailed analysis on the final submission to the COREF task was reported [7,16], of which the results of the top 4 systems are also shown in Table 1. In this analysis, the submitted predictions on the test data set of the COREF shared task are analyzed according to four types of anaphoric expressions: DNP for definite noun phrases, RELAT for relative pronouns, PRON for other pronouns including personal, possessive, and demonstrative pronouns, and OTHER for catch-all type. Examples of the coreference types are outlined below: 

“the phosphorylation status of [TRAF2] had significant effects on the ability of [the protein] to bind to CD40,” (DNP)

**Table 1:** Performance evaluation on the test data set which contains 284 protein coreference links

	**PRON (R, P, F)**	**DNP (R, P, F)**	**RELAT (R, P, F)**	**ALL (R, P, F)**
UU	12.0 79.0 20.8	5.5 66.7 **10.1**	56.0 71.2 62.7	22.2 73.3 **34.1**
UZ	17.6 62.9 **27.5**	4.1 12.5 6.2	46.7 71.4 56.5	21.5 55.5 31.0
CU	—– —– —–	—– —– —–	64.6 68.0 **66.2**	19.4 63.2 29.7
UT	12.8 72.7 21.8	1.4 14.3 2.5	29.3 73.3 41.9	14.4 67.2 23.8
RB-MIN	28.0 40.2 33.0	9.6 31.8 14.7	76.0 62.0 **68.3**	35.6 49.0 41.2
RB-MIN+1, 3	43.2 53.5 47.8	19.2 38.9 25.7	76.0 60.6 67.5	44.7 54.3 49.0
RB-MIN+1, 2, 3	48.0 50.4 **49.2**	41.1 37.0 **39.0**	76.0 60.6 67.5	52.5 50.2 **51.3**

The first four rows show the top performances in BioNLP-ST 2011, row 5, 6 and 7 are our system results with different combination of rules.

“Subnuclear fractionation reveals that there are [two ATF1 isoforms] [which] appear to differ with respect to DNA binding activity,” (RELAT)

“This ability of [CIITA] to facilitate promoter occupation is undissociable from [its] transactivation potential,” (PRON)

An analysis of the results indicated that the best resolution results for definite noun phrases (the DNP type), and several pronouns of the PRON type was 27.5% F-score and 10.1% F-score, respectively; the scores were much lower than the F-score for relative pronouns (the RELAT type), which yielded a 66.2% F-score. Thus, it can be inferred that it is more difficult to resolve definite noun phrases and pronouns than relative pronouns.

In this paper, we compare the contributions of different features in coreference resolution: discourse preference, number-agreement, and domain-specific semantic information. While discourse preference and number-agreement are two features that are often used in coreference resolution, and easy to be transferred across different domains, the use of domain-specific semantic information is varied [10,17,18]. We implemented a protein coreference system that makes use of syntactic information from the parser output, and protein-indicated information encoded in rule-based semantic classification. Experimental results showed that domain-specific semantic information is important for coreference resolution, and that simple semantic classification using semantic features helped our system to outperform the best-reported system results in the shared task.

### Related works

Several works on anaphora and coreference resolution have been carried out for the biological domain. Castano et al. [19] used a salience measure to select the best candidate antecedent. A main finding of this work is that the coercion of a verb on the semantic types of its argument plays an important role in the pronoun resolution for this domain. 46 and 54 MEDLINE abstracts were used for system development and test, respectively. Kim et al. [20] introduced BioAR, a biomedical anaphora resolution system, a system which relates entity mentions in text with their corresponding Swiss-Prot entries. This system resolves pronouns by using heuristic rules and seven patterns for parallelism. Gasperin and Briscoe [21] solved coreference resolution for full-text biological articles. This work employed a complex Naive-bayes model to train a multi-class classifer to classify the relation of expression pair. Recently, Markov Logic Network (MLN) [22,23] has been employed in Yoshikawa et al. [3] to predict coreference relation jointly with event extraction. They compared a pairwise classifier model, which is similar to Soon’s model [24], with the MLN model, and concluded that latter is better for event extraction application. However, it is difficult to fairly compare different anaphora/coreference resolution. BioNLP-ST 2011 included a task for coreference resolution with an aim to implement a fair evaluation and comparison of protein coreference resolution [7].

## Methods

In order to acquire an insight into the coreference resolution problem, we took a rule-based approach, analyzing the training data of BioNLP-ST 2011 COREF task. The performance of the system evaluated on the official test data set of the COREF task shows a significant improvement over the official winning system of the task. This section presents the overview and the performance evaluation of our system.

### System overview

**Figure 2:**
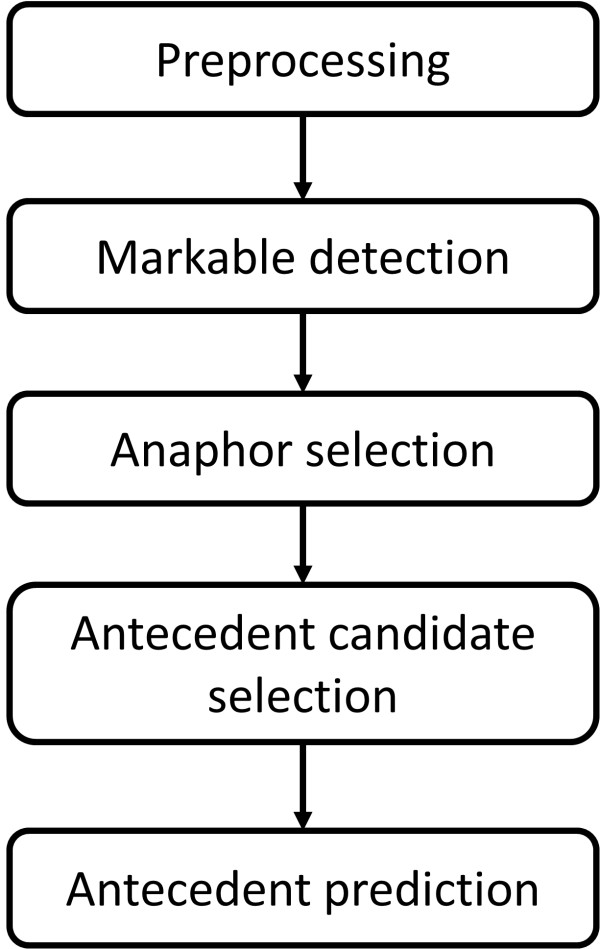
Protein coreference resolution workflow.

Figure 2 shows the overall design of the system, which includes five main components: preprocessing, markable detection, anaphor selection, antecedent candidate selection, and antecedent prediction. Processing of each component is briefly described below.

#### 

**Figure 3:**
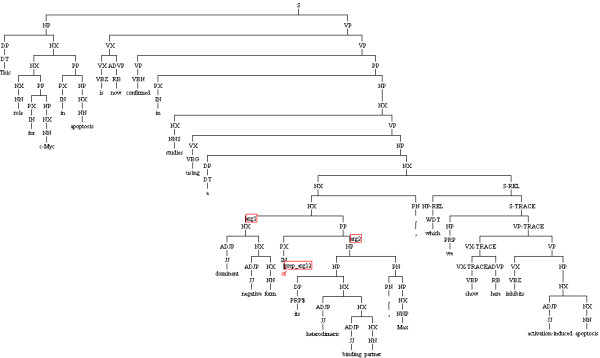
**Illustration of Enju parse output.** Enju parse output for the sentence *“This role for c-Myc in apoptosis is now confirmed in studies using a dominant negative form of its heterodimeric binding partner, Max, which we show here inhibits activation-induced apoptosis.”* The red boxes show syntactic relations between *of* and its two arguments arg1 and arg2.

**Step 0 - Preprocessing**: The input text is preprocessed using NLP tools for sentence segmentation, and syntactic parsing. We used the Genia Sentence Splitter and Enju Parser^a^[25] for sentence segmentation and syntactic parsing, respectively. Row 1 in the example of Table 2 shows three sentences as the output from the Genia Sentence Splitter, and noun phrases as the output from the Enju Parser for the sentence, S3. Due to the limited space, only a part of the phrases are shown in the table. The full parse tree for this sentence is separately shown in Figure 3.

**Table 2:** Illustration example for the system workflow of protein coreference resolution


Example text (PMID-7964516)	T cell hybridomas respond to activation signals by undergoing apoptotic cell death, and this is likely to represent comparable events related to tolerance induction in immature and mature T cells in vivo. Previous studies using antisense oligonucleotides implicated the c-Myc protein in the phenomenon of activation-induced apoptosis. This role for c-Myc in apoptosis is now confirmed in studies using a dominant negative form of its heterodimeric binding partner, Max, which we show here inhibits activation-induced apoptosis.
Preprocessing results: sentences and chunks (partially) (Step 0)	S1: T cell hybridomas respond to activation signals by undergoing apoptotic cell death, and this is likely to represent comparable events related to tolerance induction in immature and mature T cells in vivo. S2: Previous studies using antisense oligonucleotides implicated the c-Myc protein in the phenomenon of activation-induced apoptosis. S3: [This [[role] for [c-Myc] in [apoptosis]]] is now confirmed in [[studies] using a dominant negative form of its heterodimeric binding partner, Max, which we show here inhibits activation-induced apoptosis].
Markables (Step 1)	S1: [T cell hybridomas] respond to [activation signals] by undergoing [apoptotic cell death], and this is likely to represent [comparable events related to tolerance induction] in [immature and mature T cells [in vivo]]. S2: [Previous studies using [antisense oligonucleotides]] implicated [the c-Myc protein] in [the phenomenon of [activation-induced apoptosis]]. S3: [This role for [c-Myc] in [apoptosis]] is now confirmed in [studies] using a [dominant negative form of [[its] heterodimeric binding partner,[Max]]], [which] [we] show here inhibits [activation-induced apoptosis].
Anaphors (Step 2)	S1: T cell hybridomas respond to activation signals by undergoing apoptotic cell death, and [this] is likely to represent comparable events related to tolerance induction in immature and mature T cells in vivo. S2: Previous studies using antisense oligonucleotides implicated the c-Myc protein in the phenomenon of activation-induced apoptosis. S3: This role for c-Myc in apoptosis is now confirmed in studies using a dominant negative form of [its] heterodimeric binding partner, Max, [which] we show here inhibits activation-induced apoptosis.
Antecedent candidates of *its* (Step 3)	S1: [T cell hybridomas] respond to [activation signals] by undergoing [apoptotic cell death], and this is likely to represent [comparable events related to tolerance induction] in [immature and mature T cells [in vivo]]. S2: [Previous studies using [antisense oligonucleotides]] implicated [the c-Myc protein] in [the phenomenon of [activation-induced apoptosis]]. S3: [This role for [c-Myc] in [apoptosis]] is now confirmed in [studies] using [a dominant negative form of **its** heterodimeric binding partner, Max], which we show here inhibits activation-induced apoptosis.
Predicted antecedent of *its* (Step 4)	S1: T cell hybridomas respond to activation signals by undergoing apoptotic cell death, and this is likely to represent comparable events related to tolerance induction in immature and mature T cells in vivo. S2: Previous studies using antisense oligonucleotides implicated the c-Myc protein in the phenomenon of activation-induced apoptosis. S3: This role for [**c-Myc**] in apoptosis is now confirmed in studies using a dominant negative form of **its** heterodimeric binding partner, Max, which we show here inhibits activation-induced apoptosis.

The resulted items of each step are shown in square brackets, and the whole syntactic parse tree of the sentence S3 is shown in a separated figure.

**Step 1 - Markable detection**: Text chunks that are candidate coreferential expressions, which are also called *markables* following the jargon of MUC-7, are collected. For the set of markables, noun phrases, which do not include a subordinate clause, are collected as they are analyzed by a syntactic parser (in our case, Enju). Pronouns are also collected as markables. Then, for chunks that share the same head-word, which is normally the main noun of a noun phrase, only the longest chunk is taken. Since the Enju parser outputs head-word information for every noun phrase, we make use of this information for our processing, without any modification. The third row of Table 2 shows the result of markable detection for the sample text. In the sentence S3, three noun phrases recognized by the NX and NP tags of the Enju output, *role*, *role for c-Myc in apoptosis*, and *this role for c-Myc in apoptosis* (Step 0 results) share the same head-word *role*; thus, only the longest noun phrase *this role for c-Myc in apoptosis* is selected. However, between *studies* and *studies using.. apoptosis*, the former chunk is selected, since the latter contains a subordinate clause.

**Step 2 - Anaphor selection**: Candidate anaphoric expressions, which are basically pronouns and definite noun phrases are determined. A minority of anaphors are indefinite noun phrases or entity names, which act as appositions. The system first considers all pronouns and definite noun phrases in the markable set as anaphors. Then, several filters are applied to remove anaphors that are not relevant to the task definition. We implemented two types of filters: syntactic and semantic. Syntactic filters are used to filter out pleonastic its, or pronouns, like *he*, *she*, which are not expected to refer to proteins. Moreover, because our task focuses on protein references, semantic filters can be used to filter out non-protein anaphors at this stage. In practice, for definite noun phrase type of anaphors, this is accomplished, by using a list of possible head-words of protein references; for pronouns, their context words are used. More details of these methods can be found in the following section.

**Step 3 - Antecedent candidate selection**: For each anaphor, this component collects the antecedent candidates from the preceding expressions. One of the candidates will become the response antecedent, as a result of the antecedent prediction step. In theory, all expressions in the set of markables can become antecedent candidates; however, too many candidates makes it difficult to achieve correct antecedent prediction. Moreover, we also filter out candidates that violate syntactic or semantic constraints raised by the anaphor. In our system, this is done by using a particular window size in sentences, together with several syntactic filters.One of the syntactic filters is based on syntactic relations among phrases outputted from the parser. The idea behind this filter is that some types of syntactic relations imply the impossibility of coreference relations between its argument noun phrases and the inclusive expressions of these noun phrases. For example, the two expressions: *dominant negative form* and *its* in our example in Table 2, can not be coreferential with each other, since they are connected via the preposition *of*.Another syntactic filter removes pronouns that are not in the same pronoun family as the anaphor. This results in the disappearance of *this* in candidate antecedents of *its*. Pronouns in the same family as *its* are its, it, and itself.

**Figure 4:**
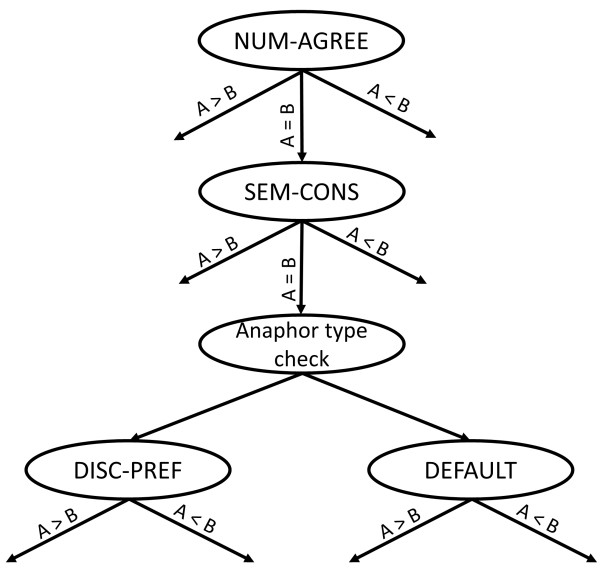
Illustration of the decision list used in antecedent prediction.

**Step 4 - Antecedent prediction**: To produce a coreference link, the antecedent candidates are sorted according to a set of preference rules, then the best candidate is chosen (Figure 4). For the sorting, the following four rules are used:

#### 

• Rule 1 (Number agreement - NUM-AGREE): The candidate, which does not conflict in number with the anaphor, is preferred.

• Rule 2 (Semantic constraint - SEM-CONS): If the anaphor is a protein reference, then a protein candidate is preferred.

• Rule 3 (Discourse preference - DISC-PREF): According to the anaphor type, the farther candidate is preferred.

• Default rule (Default discourse preference - DEFAULT): The closer candidate is preferred.

The rules are implemented using different features of expressions, such as syntactic types of expressions, head noun, semantic types, etc., in a similar way to [11]. Each rule in the decision list compares two candidates, and returns the preferable candidate in concern with the anaphor. If equility happens, the next rule in the list is applied. The default and also last rule in the decision rule list is different from the other rules in that depending on the anaphor, it prefers the closer or the farther candidate. Because of this particular rule, the decision list never results in the equility result. In this way, candidates can be sorted, and the best candidate is selected as the antecedent. Figure 4 illustrates how the decision list works when comparing two candidates: *A* and *B*.

More details concerning the implementation of the main components of our system shown in Figure 2 are presented below.

### Anaphor selection

In this step, we want to filter out those pronouns and definite noun phrases that are not a target of this task. The expressions are comprised of two types: non-anaphoric expressions, and anaphoric expressions, which do not point to proteins. The term anaphoric is used with the common sense in the NLP community. *Anaphoric expression* refers to an expression with a noun phrase as antecedent. Thus, expressions with a sentence or phrase antecedents, or nominal but successive antecedents, are not our target and should be filtered out.

Non-anaphoric expressions include first and second-person pronouns such as *I, we, you*, and pleonastic *it*. First and second-person pronouns are easily recognized by the part-of-speech tags; thus, we use part-of-speech information for the filtering. For pleonastic *it*, we make use of the following four patterns, which are similar to [26]

 It be [Adj|Adv| verb]^∗^that

 It be Adj [for NP] to VP

 It [seems|*appears*|*means*|follows] [that]^∗^

 NP [makes|*finds*|take] it [Adj]∗ [for NP]^∗^[to VP—Ving]

To recognize and filter anaphoric expressions that do not point to proteins, the system is based on the protein semantic classification results determined by the method presented below.

### Antecedent candidate selection

For each anaphoric markable, the system collects a list of antecedent candidates, and select the most probable candidate to be the antecedent of the anaphor. Basically, all of the expressions detected in the initial expression set are an antecedent candidate, with the exception of anaphoric pronouns. However, if the list contains too many candidates, then it may be more difficult for the later antecedent-selection algorithm. Therefore, candidates that are not probable to be an antecedent of the anaphor should be filtered out. There are several filters that can be used:

**Window size** Borders are set to include or exclude antecedent candidates. This is a common method for antecedent candidate filtering, as seen in the previous work [24,27,28]. Since our task focuses on anaphoric coreference, antecedent expressions normally appear relatively close to the anaphors. Thus, using window sizes is a proper technique.

**Syntactic dependency relations** Since arguments of some dependency relations (such as poss-arg12 and prep-arg12) do not corefer with each other, they can be used to correctly eliminate the number of antecedent candidates. For instance, *two such truncated forms* definitely cannot be an antecedent of *the protein* in this context: *two such truncated forms of the protein*.

### Antecedent prediction

After filtering non-relevant antecedent candidates for an anaphor in the step above, depending on the anaphor type, the remaining candidates are ranked by fixed rules, or by using a pairwise comparison procedure:

#### Fixed rules for relative pronouns

The relative pronoun can be said to be the easiest type of coreference resolution, because its antecedent expression is very close to the anaphor, and in many cases, it is right before the anaphor. For these types of anaphors, any syntactic parser can be used to find the relation between relative pronouns and their arguments. Our system accomplishes this task. It simply produces coreference links between the relative pronouns and their arguments.

However, a disadvantage to using this method is that when the parser does not find the correct arguments, the coreference also fails. This is exemplified in the following: “..of transcription factor NF-kappa B also encodes **a ****p70 I kappa Bz****protein**, I kappa *B gamma*, **which** is identical to the C-terminal 607 amino acids of..”

#### Antecedent candidate ranking based on pairwise comparison rules

This procedure compares two candidate expressions at a time with respect to preferences raised by the anaphor. The most probable antecedent expression is selected to form a response coreference link. In particular, a list of rules is used to compare two candidates of an anaphor in a deterministic manner. For each rule, both of the candidates are checked against the condition hold by the rule. If one candidate satisfies the condition and the other does not, the procedure ends with the result that the former will be preferable over the latter. If both candidates satisfy or do not satisfy the rule, the procedure proceeds to the next rule and the candidates will be checked against the condition in the same manner. The rules are applied in a successive order, one after another, until the inequality occurs, or until the end-of-the-rule list is reached. The default rule of the procedure, is in the preference of the closer antecedent candidate.

### Semantic type classification in coreference resolution

By definition, two coreferential expressions are idential, which implies a semantic-constraint on coreference relationship. In other words, semantic types of coreferents must be compatible. In practice, this compatibility is verified based on a given taxonomy of semantic classes in the following manner: two semantic classes are considered compatible or agreed with each other, when they have a synonym relation, or hypernym-hyponym relation. In this work, we only focus on the protein type, ignoring other possible semantic types, the structure of the taxonomy is not taken into account. Therefore, the likelihood that two expressions are semantically compatible, is definitely beneficial for antecedent prediction. Focusing on specific entity types, i.e., protein type, enables us to find a proper method for determining the likelihood, and method for encoding the likelihood in coreference resolution.

#### Accurate semantic type classification based on given Pronoun mentions, for nominal expressions (ANTE-SEM)

Since gold-standard (gold, hereafter) protein annotations are provided as input to the task, we can use them in combination with syntactic information to judge whether an expression is a protein-referential expression or not. If an expression is a noun phrase with a single head-word, and it contains a protein mention that completely overlaps with the head-word, then the expression is classified as a protein reference. In another case, when the head-noun of an expression is either *protein* or *gene*, and has a protein mention as its premodifier, such as *the Tax protein*, the expression is also a protein reference. For a coordinated noun phrase, if one of its constituents is classified as protein, then that noun phrase is also classified as protein.

#### Semantic type classification for pronominal anaphors (PRO-ANA-SEM)

Pronouns, in particular, possessive pronouns, occupy the majority of anaphoric pronouns in biological texts (Table 3). However, they do not contain in themselves very much useful information for the resolution; thus, we need to acquire more information from its context [29]. The analysis of BioNLP-ST 2011 also showed that we need a different strategy to resolve such pronouns [16]. Fortunately, the key to this problem lies in the context of pronouns.

**Table 3:** Anaphor types in descending order of percentage measured on the training data set

**Type**	**Percentage**	**Number**	**Examples**
Possessive pronoun	25.3	222	*its, their*
Relative pronoun	18.6	163	*that, which*
Demonstrative noun phrase	15.2	133	*these genes*
Demonstrative pronoun	13.6	119	*this, those*
The- definite noun phrase	10.9	96	*the protein,this factor*
Personal pronoun	9.6	84	*it, they, them*
Other definite noun phrase	2.2	19	*both-NP*
Indefinite noun phrase	1.6	14	*a factor*
Proper name	1.5	13	*IL-2*
Other pronoun	0.8	7	*both, either*
Reflexive pronoun	0.8	7	*itself*
Total		877	

We implemented a simple function to classify the semantic type of a possessive pronoun, based on its context word. In particular, we check the noun phrase in which the determiner is *its* or *their*; if the noun phrase contains a *protein key word* then the inclusive pronoun is classified into the protein semantic type. *Protein key words* can be a verb, a noun or an adjective that co-occurred with protein mentions, and can be used as a clue to distinguish the protein type from other semantic types. For example, the word *binding* in the following noun phrases: *its heterodimeric binding partner*, or *its binding site*, is a clue to infer that *it* must be a protein reference. For our preliminary experiment, we collect these key words manually by checking the noun phrases containing *its* and *their* in the training data. Our final protein key word set includes 12 words: *binding*, *expression*, *interaction*, *regulation*, *phosphatase activity*, *localization*, *gene*, *sequence*, *region*, *phosphorylation*, *transactivation*, and *transcription*. In future, the protein key words can be collected automatically using the term corpus, or other resources of proteins.

#### Semantic type classification for definite noun phrase anaphors (DEFNP-ANA-SEM)

Definite noun phrases do not always have an antecedent in textual context. In particular, in biomedical scientific papers, many definite noun phrases do not have antecedents, since the referenced concepts can include any concept that is understood by subject matter experts in the domain. Distinguishing such *non-anaphoric* definite noun phrases from *anaphoric* ones is a difficult task. Knowing their semantic type helps to filter out irrelevant candidate antecedents, thereby increasing the chance of picking up the right antecedent, and increasing the precision of antecedent prediction.

In our implementation, the decision to keep an anaphoric expression for further processing steps for an anaphoric definite noun phrase is based on a protein head word list. We tested two different head word lists: one is built automatically from the gold anaphoric nominals in gold data; the other word list contains the top seven common head words: *protein, gene, factor, molecule, element, family, inhibitor*, and *receptor*. Using this head-word list and premodifiers, the system covers 83.5 percent of the coreference links.

#### Encoding semantic types in coreference resolution

Semantic type information can be used in coreference resolution in several ways. First, in anaphor selection, semantic information can be used to filter out non-protein anaphoric expressions. Second, in antecedent candidate filtering, semantic agreement between the antecedent candidates and the anaphoric expression is verified. Those candidates that are not in agreement with the anaphor in semantics are filtered out. For example, if an anaphor is classified as a protein referent, then the non-protein antecedent candidates are removed from the candidate set of the anaphor. Finally, in antecedent prediction: semantic agreement can again be used as a constraint when comparing two antecedent candidates to select the more probable candidate.

## Results

### Performance evaluation

Our minimal system configuration RB-MIN includes all of the processing and filters from step 0 to step 3, as explained in the Methods section. To keep the minimal configuration simple, step 4 - antecedent selection of the baseline only uses the default comparison rule, which assures that the closest antecedent candidate is selected. For antecedent candidate selection, the window size used in step 4 is set to 2, which means that antecedent candidates are collected in the two nearest sentences from the anaphor, and the sentence embedding the anaphor. The statistics measured on the training set of the corpus shows that 97.0% percent of protein coreference links have antecedents appearing in within 2 sentences. With this window size, the average number of candidates per anaphor is 6.1. Also, experiments with wider window sizes did not improve performance.

Table 1 compares our system with the top four official results of the COREF shared task in BioNLP-ST 2011 [16]: UU [13], UZ [30], CU, and UT [31]. The scoring scheme used throughout this paper is the protein coreference evaluation, the primary evaluation method of the COREF shared task [16]. This primary evaluation method, which was particularly designed for the shared task, is based on protein coreference links that have been automatically generated from manually annotated coreference links. The last column *ALL* shows the overall results, while its preceding three columns *PRON*, *DNP*, and *RELAT* shows the protein resolution results by three major subtypes of anaphors: pronouns, definite noun phrase and relative pronouns, respectively. Note that the results from RB-MIN with minimal configuration already surpasses the best results obtained by the UU team, with up to 7.1% higher performance in F-score. Since RB-MIN uses similar preprocessing tools as UU [13], but less information in antecedent prediction, this gap in performance is likely caused by the different markable detection methods. UU pointed in their paper that markable detection is one of the challenges of this task [13]. In their system, UU used a machine learning approach, and tested two distinguished models for markable detection: one solved both anaphors and antecedents together, the other treated anaphors and antecedents separately. Meanwhile, our method is basically based on the boundary of noun phrases and pronouns, as is outputted from the parser. The patterns used to extract the proper noun phrases and pronouns, are manually designed in relation to the markable boundaries annotated in the training data.

Breaking down the system performance by the different types of anaphors provides us with insight into what has been accomplished/solved by our methods, and also provides us with improvement opportunities. Concerning the RELAT type of coreference, we can see that RB-MIN and RB-FULL both achieve comparable results with the best-performing team in BioNLP-ST 2011. However, it should be noted that our antecedent prediction for the RELAT type is based solely on the output of the Enju parser for the RELAT type, so in order to improve this type of coreference, we have to find ways to overcome the parse errors on noun phrase boundary detection and relative clause attachment (see Section Discussions).

The increase in system performance on the PRON and DNP types by RB-FULL demonstrate the effectiveness of discourse and semantic information in the performance of protein coreference resolution. Comparing RB-MIN, RB-FULL and RB-MIN+1, 3, we found that rule 3, which stands for discourse preference, works well for the PRON type (2). On the other hand, the major contribution to the improvement of DNP resolution is from rule 2. This rule successfully utilizes the domain-specific information, which shows that coreference resolution requires domain-specific information. To further explore the elements contributed to this significant improvement, we analyzed our system in more detail. The analyses of the results are provided in the section entitled Discussions.

## Discussions

### Contribution of rules

Table 4 compares various configurations of our system. The first row in the table, RB-MIN, is the minimal configuration of the system. The following three rows show contribution of the three rules, NUM-AGREE, SEM-CONS, and DISC-PREF. RB-FULL is the full system. To emphasize the contribution of the semantic rules, it also shows RB-FULL-sem system.

**Table 4:** Contribution of different antecedent prediction rules in coreference system

**EX**	**R**	**P**	**F**
RB-MIN	37.7	61.6	46.8
RB-MIN + 1 (NUM-AGREE)	39.7	64.8	49.2
RB-MIN + 2 (SEM-CONS)	47.1	58.2	52.0
RB-MIN + 3 (DISC-PREF)	46.6	65.1	54.3
RB-MIN + 1, 2, 3 (RB-FULL)	57.8	67.8	**62.4**
RB-MIN + 1, 3	50.5	69.1	58.4

Performance was measured on the development data set which contains 204 protein coreference links.

The full combination of rule 1, 2 and 3 resulted in a 62.4% F-score (RB-MIN+1, 2, 3) (Table 4). In this full configuration, rule 2 contributes a 4-point F-score increase in the development set, and 2.3-point F-score increase on the test set, when comparing RB-MIN+1, 3 and RB-MIN+1, 2, 3. However, the result of RB-MIN is still more than 7 points higher than in state-of-the-art performance. This gain is due to the fact that the rule ensures that the semantic type of antecedents is the same as for their anaphors, thus enabling the correct detection of antecedents. In other words, if an anaphor is classified as a protein reference, then the antecedent must also be a protein reference. The following examples illustrate the way rule 2 works. 

• “Therefore, [IRF-1] may be an important contributor to IL-12 signaling, and we speculate that the defective IL-12 responses seen in IRF-1-/- mice might be attributable, in *part*, to the absence of [this transcription factor].”^b^ (PMID-10358173)

Coreference examples in this paper are represented as below: gold anaphoric and antecedent expressions are bracketed, antecedents before anaphors; gold protein mentions are underlined; and incorrect response antecedents are in italics.

In the example above, without rule 2, the faulty response antecedent of *this transcription factor* is *part* because it is the closet antecedent candidate agreeing with the anaphor on the singular number. Meanwhile, since *this transcription factor* is recognized as a protein reference, its closest protein antecedent *IRF-1* was successfully detected by RB-FULL.

Another example is: 

• “This role for [c-Myc] in *apoptosis* is now confirmed in studies using a dominant negative form of [its] heterodimeric binding partner, Max, which..” (PMID-7964516)

Concerning the anaphoric pronoun *its* in this example, there are several antecedent candidates: *this role*, *c-Myc*, *apoptosis*, *studies*, *a dominant negative form of its heterodimeric binding partner*. Although *studies* and *a dominant negative form of its heterodimeric binding partner* have been crossed out because of disagreement in numbers, and violation of abandoned syntactic constraints, correspondingly, the system would return the incorrect antecedent *apoptosis* instead of *c-Myc*. Fortunately, the containing noun phrase of the anaphor *its* has the modifier word *binding*, which is a clue for classifying *its* as a protein reference (See Semantic type classification for pronominal anaphors (PRO-ANA-SEM)). Rule 2 utilizes semantic classification result to make the correct selection.

### Contribution of semantic information in anaphor selection

In our system, domain-specific semantic information is utilized in two places: anaphor selection and antecedent prediction. The effect of semantic information in antecedent prediction has been analyzed in the sections above. In this subsection, we are going to explore the contribution of semantic information in the anaphor selection step.

To classify anaphors into protein or non-protein reference, our system employs a head-word based classifier for definite noun phrases, DEFNP-ANA-SEM, and a context-based classifier for pronouns, PRO-ANA-SEM (Section Methods). Without limiting the number of anaphors by using semantic information-based filtering, the precision significantly drops, causing a big decrease in the F-score (Table 5, RB-FULL without DEFNP-ANA-SEM). This decrease is due to the fact that the semantic filter is the only method for filtering out definite noun phrase anaphors. Without the filter, all definite expressions, which include a substantial amount of non-anaphoric expressions, are considered as anaphors. Besides the anaphoric use, definite noun phrases are also used to refer to entities or concepts in the common domain knowledge shared between readers and writers. Statistics in [32] show that only around 30% of definite noun phrases are anaphoric, and the other uses according to their classification include associative, unfamiliar/larger situation, idiom and doubt. Distinguishing such non-anaphoric definite noun phrases from anaphoric ones is extremely difficult.

**Table 5:** Influence of semantic information used in anaphor selection step on coreference resolution system

**EX**	**R**	**P**	**F**
RB-FULL	57.8	67.8	62.4
RB-FULL w/o PRO-ANA-SEM	55.4	66.9	60.6
RB-FULL w/o DEFNP-ANA-SEM	55.9	14.5	23.0
RB-FULL w/o DEFNP-ANA-SEM + PRO-ANA-SEM	53.4	13.9	22.1

Performance was measured on the development data set which contains 204 protein coreference links.

In our system, contextual information of possessive pronouns is utilized through the protein key words (Section Methods), and this contributed to a 1.8% gain in F-score (Table 5, RB-FULL without PRO-ANA-SEM). This gain is a good indication for seeking a systematic method to develop and include such contextual information in coreference resolution. Examples showing the effectiveness of semantic information from the context of pronouns is provided below: 

• “This role for [c-Myc] in *apoptosis* is now confirmed in studies using a dominant negative form of [its] heterodimeric binding partner, Max, which..” (MID-7964516)

• “This ability of [CIITA] to facilitate *promoter occupation* is undissociable from [its] transactivation potential.” (PMID-10221658)

• “In transient transfectin experiments, [BCL6] can repress *transcription from promoters linked to [its] DNA target sequence* and this activity is..” (PMID-8692924)

• “[Human immunodeficiency virus type 1 (HIV-1) Tat], an early regulatory protein that is critical for viral gene expression and replication, transactivates the HIV-1 long terminal repeat (*LTR*) via [its] binding to the transactivation response element (TAR) and,..” (PMID-9261367)

In all the examples above, the appearance of words such as *binding*, *transactivation*, *DNA target sequence* in the noun phrases for which the anaphor plays a role as a determiner, is a contextual indicator for the protein type. Since the anaphors are predicted as protein reference from their context, the system correctly detects their protein antecedents.

### Comparing with the existing scoring schemes

So far, several scoring schemes have been proposed to evaluate coreference resolution. The MUC score [14] is based on the assumption that coreference links effectively partition the entity references. The precision, recall, and F-score are then calculated based on the minimal number of the required insertion or deletion operations on the coreference links to transform the partition by the response coreference links to the one by gold links. Bagga and Baldwin [33] proposed the B-cubed (B3) score, which is calculated based on the individual coreference links, not the resulting partitions. Luo proposed the CEAF score which is calculated based on the best mapping between coreference expressions or entities, thus results in two types of CEAF: expression-based (CEAF-M) and entity-based (CEAF-E) [34]. The best expression mapping used in CEAF is found using Kuhn-Munkres algorithm. Recently, the BLANC score was introduced [35], to be used for the CoNLL coreference resolution shared task [36]. BLANC makes use of a clustering evaluation metric called Rand index [37] to measure the similarity between two partitions of coreference expressions.

We compared the BioNLP-ST scoring scheme with the existing schemes mentioned above. The scores for MUC, B3, CEAF-M, CEAF-E, and BLANC were achieved using the scorer of CoNLL shared task [36]. The comparison result is shown in Table 6. It is observed that the BioNLP, BLANC and CEAF scores are relatively similar to each other, which makes sense considering that all the three are calculated based on the number of individual coreference links in the response and gold links. On the other hand, the MUC score is much lower, and the B3 score is much higher than the BioNLP-ST score. These results agree with the criticisms on the shortcomings of MUC-score and the looseness of B3 [38].

**Table 6:** Evaluation of RB-FULL system with different coreference evaluation scores

**Score**	**R**	**P**	**F**
BioNLP-ST	57.8	67.8	**62.4**
MUC	28.9	32.4	30.5
B3	72.9	77.2	75.0
BLANC	61.3	63.4	62.2
CEAF-M	68.2	68.2	68.2
CEAF-E	66.6	62.5	64.5

Performance was measured on the development data set which contains 204 protein coreference links.

### Other issues

**Other challenges specific to the protein coreference task** Number agreement is a constraint in English writing. However, in the data, we found several coreferential expressions that violate this constraint. The anaphor and antecedent in the following is an instance of this violation: 

• “..for OTF-2 in DRA gene transcription. In contrast, [OTF-1-enriched protein fractions] did not affect DRA gene transcription although [it] functionally enhanced the transcription of another..” (PMID-1560002)

**Coreference annotation and evaluation** Therefore, when the proteins appear in premodifiers or postmodifers of noun phrases as [cDNAs encoding EBF or a covalent homodimer of E47] in the following example, such proteins might not be the correct antecedent proteins. 

• “With the aim of identifying genetic targets for these transcription factors, we stably transfected [cDNAs encoding EBF or a covalent homodimer of E47], *individually or together*, into immature hematopoietic Ba/F3 cells, which lack [both factors].” (PMID-9252117)

**Parse error** Coreference expression boundary is determined mostly based on noun phrase boundary output from the parser. Therefore, parse error on noun phrase boundary strongly affects the performance of coreference resolution. Examining the data, we found that many antecedent expressions of plural anaphors are coordinated noun phrases, which are unfortunately difficult cases to many parsers including Enju. Incorporation of recent works for coordination resolution like [39] should be useful for improving the performance of the parser. The following example shows a coordination-structured antecedent *AML1/CBF beta, C/EBP, Ets, c-Myb, HOX, and MZF-1* that failed to be detected by the parser. The spurious response expression is *transcription factors from several families*. 

• “granulocytic and monocytic lineages, *transcription factors from several families* are active, including [AML1/CBF beta, C/EBP, Ets, c-Myb, HOX, and MZF-1]. Few of [these factors] are expressed exclusively in myeloid cells;..” (PMID-9291089)

## Conclusions

From the results of the BioNLP-ST COREF task, it is analyzed that the use of semantic information is necessary to improve the performance of protein coreference resolution. This paper presented an improvement of protein coreference resolution by particularly using domain-specific semantic information. Other popular techniques for coference resolution, e.g., number agreement checking and distance-based preference, were also implemented and tested.

Experimental results show that those techniques can improve the coreference resolution performance significantly. Nevertheless, the current performance is still far from satisfaction (50.2 % precision, and 52.5% recall), and there is a much room for improvement. Future works include more elaborate acquisition and use of semantic knowledge.

## Endnotes

^a^Enju parser comes with a default tokenizer and part-of-speech tagger for biological text.

^b^Coreference examples in this paper are represented as below: gold anaphoric and antecedent expressions are bracketed, antecedents before anaphors; gold protein mentions are underlined; and incorrect response antecedents are in italics.

## Competing interests

The authors declare that they have no competing interests.

## Authors’ contributions

All authors contributed to the production of the manuscript. JDK, MM, TM and TJ supervised all steps of this work. NLTN built the system and carried out the experiments. All authors read and approved the final manuscript.
